# Optimizing efficiency in the creation of patient-specific plates through field-driven generative design in maxillofacial surgery

**DOI:** 10.1038/s41598-023-39327-8

**Published:** 2023-07-26

**Authors:** Alessandro Tel, Erik Kornfellner, Francesco Moscato, Shankeeth Vinayahalingam, Tong Xi, Lorenzo Arboit, Massimo Robiony

**Affiliations:** 1 Maxillofacial Surgery Unit, Department of Head-Neck Surgery and Neuroscience, University Hospital of Udine, Udine, Italy; 2 Center for Medical Physics and Biomedical Engineering, Medical University of Vienna, Vienna, Austria; 3 Ludwig Boltzmann Institute for Cardiovascular Research, Vienna, Austria; 4 Austrian Cluster for Tissue Regeneration, Vienna, Austria; 5 Department of Oral and Maxillofacial Surgery, Radboud University Medical Center, Nijmegen, The Netherlands; 6 Sant’Anna School for Advanced Studies, University of Pisa, Pisa, Italy; 7 Maxillofacial Surgery Department, Maxillofacial Surgery Unit, Academic Hospital of Udine, Department of Medicine, University of Udine, P.le S. Maria Della Misericordia 1, 33100 Udine, Italy

**Keywords:** Image processing, Computational models, Medical research, Biomedical engineering

## Abstract

Field driven design is a novel approach that allows to define through equations geometrical entities known as implicit bodies. This technology does not rely upon conventional geometry subunits, such as polygons or edges, rather it represents spatial shapes through mathematical functions within a geometrical field. The advantages in terms of computational speed and automation are conspicuous, and well acknowledged in engineering, especially for lattice structures. Moreover, field-driven design amplifies the possibilities for generative design, facilitating the creation of shapes generated by the software on the basis of user-defined constraints. Given such potential, this paper suggests the possibility to use the software nTopology, which is currently the only software for field-driven generative design, in the context of patient-specific implant creation for maxillofacial surgery. Clinical scenarios of applicability, including trauma and orthognathic surgery, are discussed, as well as the integration of this new technology with current workflows of virtual surgical planning. This paper represents the first application of field-driven design in maxillofacial surgery and, although its results are very preliminary as it is limited in considering only the distance field elaborated from specific points of reconstructed anatomy, it introduces the importance of this new technology for the future of personalized implant design in surgery.

## Introduction

Contemporary oral and maxillofacial surgery increasingly incorporates customized devices created using the patient’s anatomy as a guiding template. Hence, customized devices provide a natural and precise fit with the bone, offering advantages in terms of easy placement, reduced surgical time, and increased surgical accuracy^1–3^.

The advent of additive manufacturing (AM) in the healthcare field provided a strong impulse to the immediate translation of designed shapes into 3D printed implants, extending the concept of personalization to a growing number of surgical scenarios. The design of personalized 3D-printed implants is still an open issue for maxillofacial surgery devices and represents one of the main reasons to search for newer strategies specific for AM. Moreover, progresses in computerized simulations, including finite element analysis (FEA), led to improved reliability of personalized implants, which can undergo a virtual biomechanical testing by applying defined forces, boundaries, and material properties to predict critical strain areas that might be subject to failure, allowing to improve the implant shape before it is manufactured^4–6^.

The design of these devices generally involves using computer-aided design (CAD) software to model the final object in an ordered sequence of 3D modeling operations, starting from an empty shape and using the underlying anatomy as a reference. This process is conventionally referred to as “explicit modeling”. It resembles an engineering drawing process and results in a mesh with tessellation and topology defined by the user and the sequence of implemented design operations.

Recently, new software packages have implemented sophisticated algorithms to represent implicit geometry. Mathematically, an implicit surface is defined by a continuous volume function F(x, y, z) = 0 at an infinite level of detail, which implies the surface exists within the mathematical function. The surface is said to be implicit when the equation is not solved for x, y, and z. The geometry becomes explicit when the equation is solved, and an approximation of that surface is represented as a triangulated wireframe (mesh). Implicit modeling relies solely on volume functions, making it a powerful tool to define, change, and represent 3D geometry without directly rendering a complex polygonal network of vertices, edges, and faces. Thus, implicit bodies are significantly lighter to compute and maintain their pure form since they are not discretized into geometrical subunits, which fail to accurately represent the surface continuity at the cost of demanding computational usage. Implicit bodies can be considered entities related to a value within each point of the 3D space. This is similar to how fields are used in physics to define continuous variations of quantities such as temperature, electromagnetism, or flow. For design purposes, the scalar field defines implicit bodies, the gradients of 3D geometry. Currently, nTopology (nTopology Inc., New York, NY, USA) is the only engineering design software that permits a Field-Driven Design approach, providing enhanced flexibility for various complex applications, including the computationally demanding generative design and lattice structures^7^. While the field-driven generative design has been used to various extents in engineering literature, little is known about its application in healthcare^8–10^. To date, there have been no reports of any potential surgical applications, and this approach has not been implemented in oral and maxillofacial surgery specifically.

This paper aims to assess whether field-driven design can be a feasible method to generate customized osteosynthesis plates for orthognathic surgery and facial traumatology using the nTopology software in conjunction with the current workflow of computer-guided surgery. The authors also investigated the implications of field-driven generative design (FDGD) in creating patient-specific maxillofacial implants, particularly in terms of time reduction and automation of the design process.

## Materials and methods

### Study design

This study was conducted between November 2022 and March 2023. It is a non-clinical, in-silico, retrospective longitudinal observational study, which included anonymized models of patients operated on at the University Hospital of Udine. Procedures considered included orthognathic surgery and traumatology. Given this study's non-clinical design and speculative purpose and considering that simulations were performed on anonymized virtual models and not translated into clinical practice, ethical approval was not required.

Analyses were performed using the following hardware: Apple iMac Pro (2017), OS: Microsoft Windows partitioned using Bootcamp, 32 GB RAM, CPU Intel Xeon W a 3.2 GHz 8‑core, GPU Radeon Pro Vega 56 with 8 GB VRAM.

This study included models of 10 patients undergoing conventional bimaxillary orthognathic surgery and 10 patients with craniofacial trauma. Orthognathic surgery was performed either using standard plates (5 patients) or patient-specific osteosynthesis plates (5 patients), the latter option being preferred in cases with multiaxial skeletal movements, including translations and rotations performed in x, y and z axes. To standardize the procedure, trauma cases were limited to zygomatic-maxillary complex (ZMC) fractures and Le Fort I fractures. Trauma cases were all operated using standard stock plates. Based on the type of plates used and their diagnosis, patients were subdivided into three groups: group 1 included orthognathic surgery performed with stock devices, group 2 included orthognathic surgery performed using custom devices, and group 3 included trauma surgery operated using stock plates.

Surgical planning for the procedure was performed in 3-Matic software (Materialise, Leuven, BE) and project files were exported respectively in the final planning position for orthognathic surgery, namely in the final position of the bimaxillary complex and in the fully-reduced fracture fragments configuration for trauma surgery (Fig. 3).

### Model preparation

To enhance model correspondence, all geometries were prepared in 3-Matic (Materialise, Leuven, BE) using a re-meshing algorithm with the following parameters: adaptive re-mesh, shape measure: skewness, shape quality threshold: 0.836, maximum geometrical error: 0.050, preservation of surface contours: enabled.

To facilitate subsequent processing within the software for implicit modelling, all subcomponents of processed geometries were merged within a single entity, and a wrapping operation was applied to generate a single, simplified geometry with the following parameters: gap closing distance: 5.000; smallest detail: 0.300; resulting offset: 0, preservation of surface structure: enabled. The importance of wrapping was to create an unified geometry devoid of surface interruptions, such as fracture lines, osteotomies or segmented geometry hollowing, that could have hindered the proper functioning of the implicit modelling algorithm. Moreover, the wrapping operation was essential for simplifying geometries by consolidating them into a single external shell, thereby preventing many mesh errors, such as inverted normals and overlapping triangles. Re-meshed and wrapped models were exported as binary STL files.

### Definition of screw position

As in several conventional design approaches, plate creation started from the definition of screw location. Rather than placing cylinders to represent the screws, the authors only defined a point (analytical primitive) placed on the geometry, which defined the theoretical position of the future screw hole of the plate. Points were placed according to surgical guidelines over the strain-bearing pillars of the maxillofacial skeleton and regions with maximal bone thickness. A map of the placed points was exported as an XML file. As nTopology requires, inputs provided as a scalar point map were formatted as X, Y, and Z. Subsequently, the XML file was converted into a CSV file. A comma separated each coordinate value.

### Field-driven generative design of patient-specific plates

STL of wrapped and remeshed parts were imported in nTopology software, an innovative software enabling field-driven engineering design. Being capable of representing 3D objects within a field described by mathematical equations rather than by geometrical entities, nTopology can handle extremely complex geometries with reduced computational usage. The necessary step involved converting geometrical vertex-based entities into implicit bodies based on distance fields using the “Implicit Body from Mesh” script. The Bone Plate Generator module performed a generative, conformal plate design. First, the screw hole location was defined by importing the scalar point map in CSV file format, allowing to display of the points set in 3-Matic on the implicit body. The following values parametrically described plate features: concerning plate holes, flange size, flange depth and fillet radius were respectively fixed at 0.5 mm, 0.5 mm and 0.05 mm; concerning bone plate specifications, plate thickness, rim offset, and fillet radius varied depending on the anatomical site and the type of surgical procedure. After such parameters were defined, the bone plates were generatively created around the predefined screw holes, covering a variable area over the maxillofacial skeleton. Modification of the blend radius enabled changes to the total bone area coverage around the predefined holes, making the plate smaller or larger, depending on the required extension across the fitting surface.

**Figure 1:**
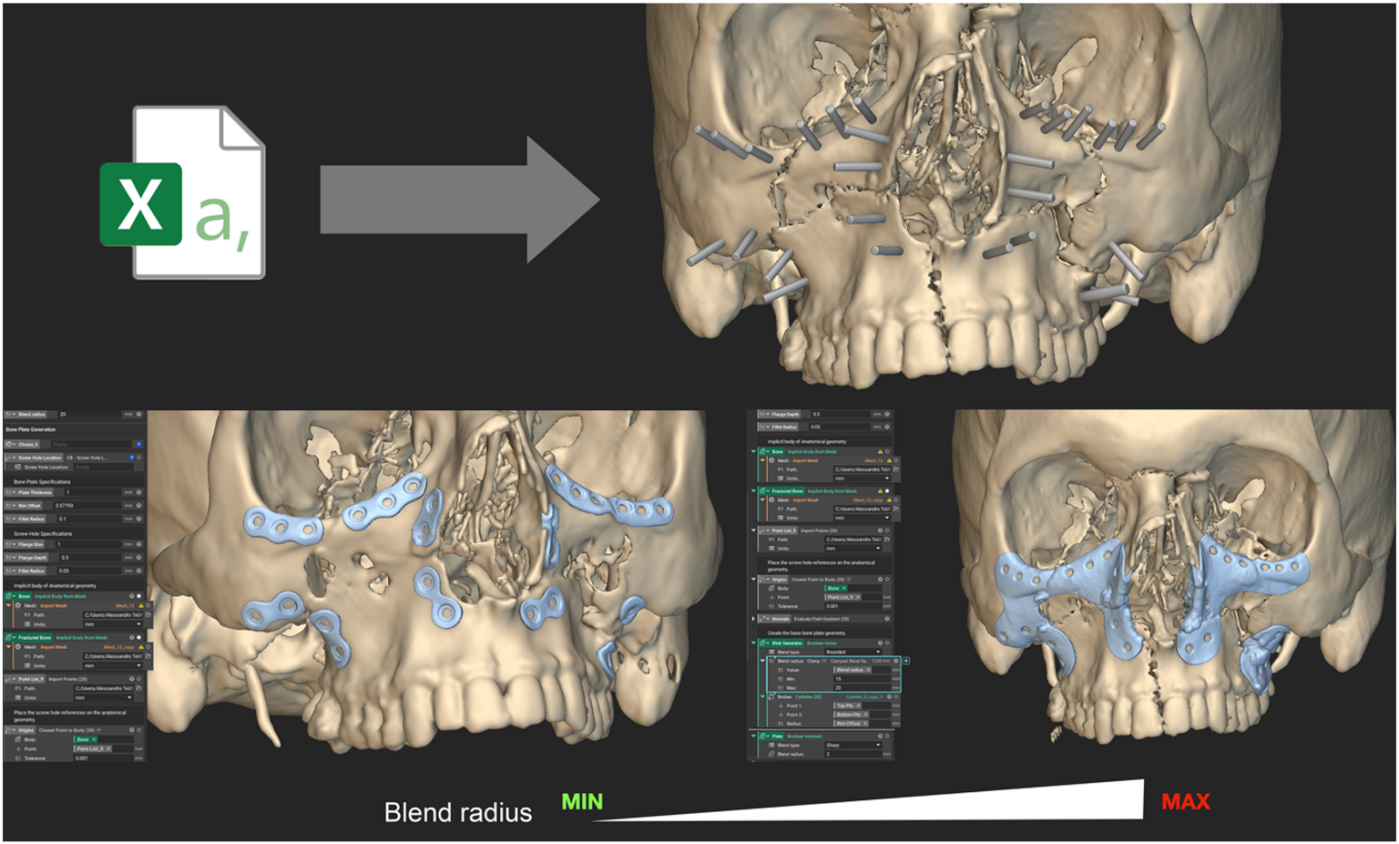
Working phase in nTopology for a combined ZMC-Le Fort I fracture (group 3). Defining the primitives (i.e. the points) allows to compute screws and design the plate automatically. Increasing the blend radius extends the total coverage area around the screw holes.

Figure 1 represents the creation of customized plates in case of trauma surgery starting from an imported scalar point map, with varying degrees of blend radius, to determine the plate coverage around screw holes.

### Remodeling of generatively-designed plates

Once all plates were successfully generated, a script to convert the implicit body into tessellated geometry with an error threshold of 0.1 mm was applied. The resulting mesh was exported as an STL file. STLs were subdivided into two groups:

- When points were placed close to each other, the modification of the blend radius yielded a unique plate.
- When points were placed in contiguous groups, but with a spaced region, the modification of the blend radius yielded the fitting surfaces of the plate’s screw-bearing region, which we name “flange”.

**Figure 2:**
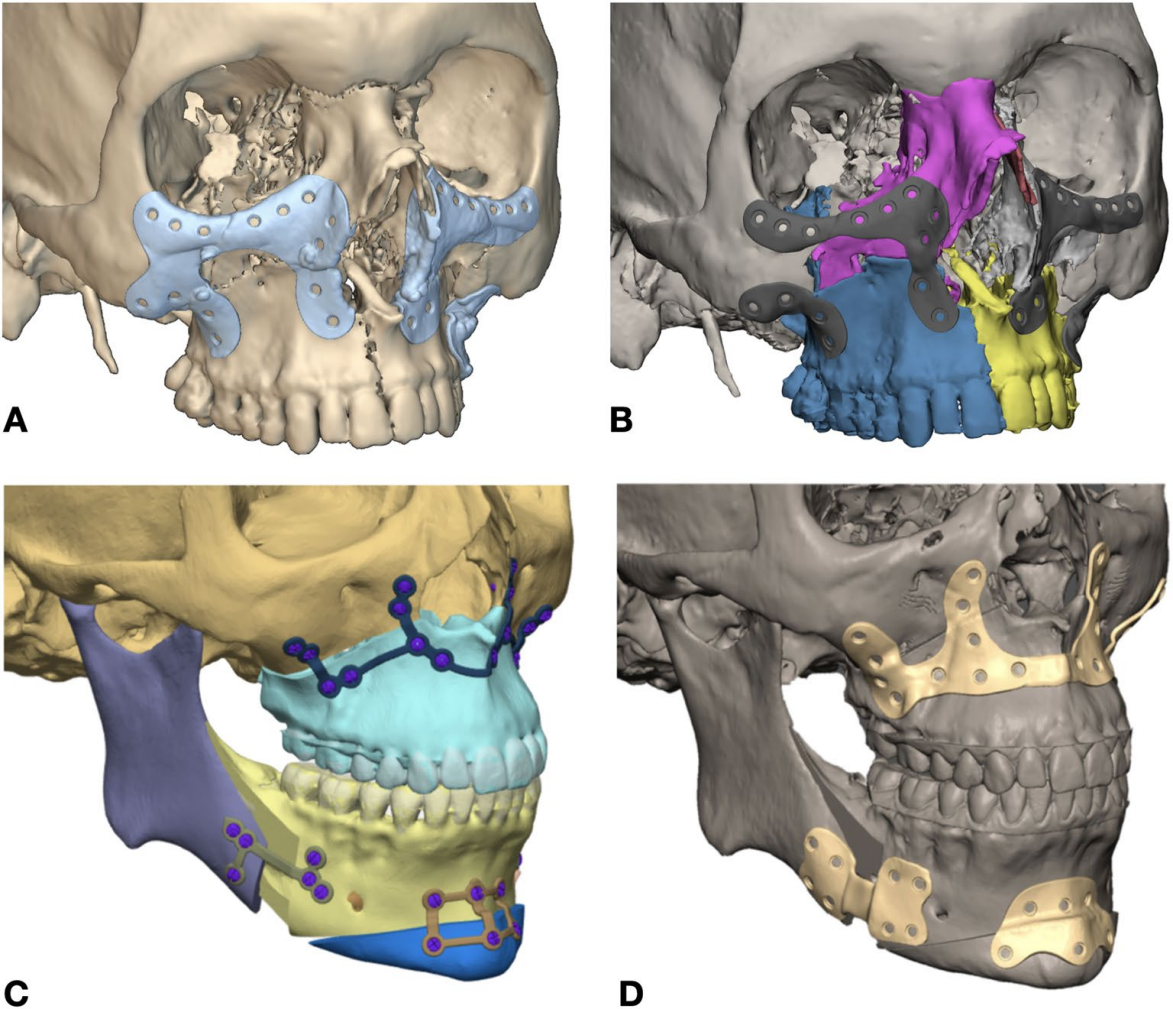
(**A**, **B**) implants determined by generative design within the scalar field (implicit bodies) are shown in the left panel for a class 3 patient. After trimming, separate plates functional for surgical purposes can be achieved (right panel). (**C**, **D**) implants used during surgery for a class 2 patient and produced by a certified medical manufacturer (**C**), and comparison with the corresponding primary implant shape designed using an FD-based workflow after postprocessing (**D**).

**Figure 3:**
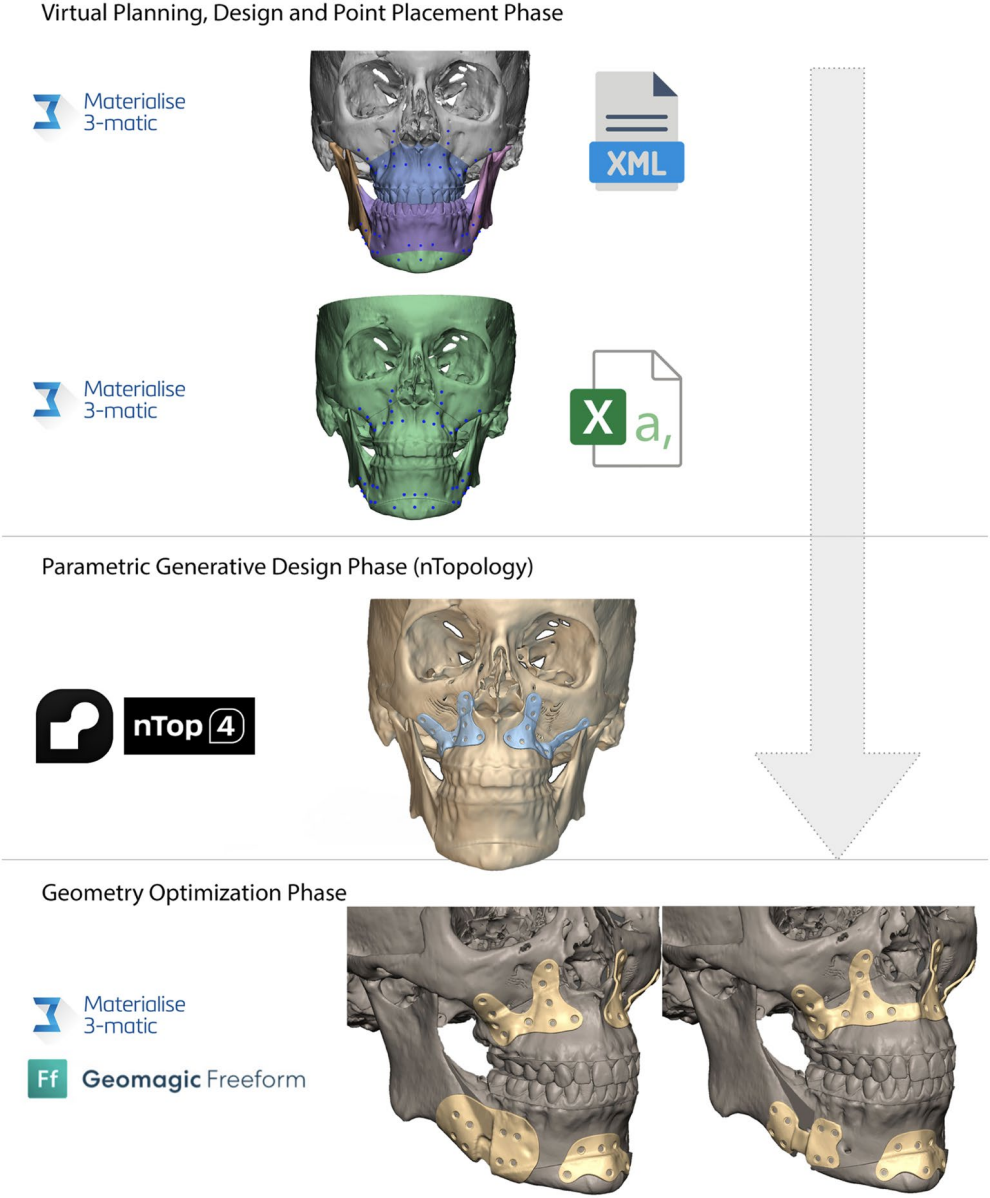
The graphical workflow shows how field-driven generative design can be integrated into cranio-maxillo-facial surgical planning.

STLs of plates and flanges were imported in 3-matic or Geomagic Freeform (3DSystems, Rock Hill, SC, USA) and overlapped with the original planning to determine which regions needed to be trimmed off or remodeled according to the surgical needs (Fig. 2). While 3-matic is more specific for traditional geometry operations including Booleans and spline design and is usually referred as an anatomical-CAD package, Freeform is more suitable for organic modeling, thus the combination of both represents a valuable strategy to postprocess preliminary implant shapes. As an example, when creating mandible plates, it was essential to include a notch around the mental nerve to prevent compression injuries. After achieving separate flanges, a reuniting trait or a “bridge”, was created in the design software to form a single plate. Figure 3 summarizes the entire flow for creating customized orthognathic surgery plates.

### FEA testing of implants

To perform the FEM analysis, a volume mesh was created in nTopology with a maximum mesh edge length of 1 mm and a growth factor of 2. The screws were represented by tie constraints at the corresponding screw positions. TI–6Al–4V (with a Young's modulus of 113.8 GPa) was assumed for the bone plates, while the cortical bone was modelled isotropically with a Young's modulus of 20 GPa.

For the genioplasty application, the chin was separated from the skull. A force of 40N was applied according to the anterior digastric muscle^11^. The top of the skull was fixed with a displacement restraint.

In the BSSO (Bilateral Sagittal Split Osteotomy) applications, the parts were additionally connected above the osteotomic gap region. The anterior part of the implant was loaded with 20 N per screw in the occipital direction.

For the simulation of the Le Fort I plate, only the forces of the two masseter muscles were used. For this simulation, the mandible was removed and the teeth of the maxilla were assumed to be fixed. A force of 350 N was applied to both zygomatic arches in the direction of the masseter muscle.

### Evaluation of performance of nTopology

Performance of nTopology compared to the conventional design strategy for the creation of a patient-specific craniomaxillofacial plate was evaluated by comparing the time spent in the design of the corresponding implant. The manufacturer was asked to provide the time sent by an expert clinical engineer to design a craniomaxillofacial plate using a conventional CAD software. This time was compared with the time spent to design a similar implant based on implicit modeling using nTopology. The evaluation was separately conducted for orthognathic surgery cases, considering the Le Fort I plate, both BSSO plate and genioplasty plate, as well as for trauma cases, including a customized plate for Le Fort I and ZMC fracture patterns. The evaluation of time in nTopology included the definition of point coordinates, calculation time within the software, and postprocessing of the primary implant shape by trimming and remodeling of inappropriate areas of the plate.

Statistical evaluation was conducted using Stata software (Stata Corporation, College Station, TX, USA). A two-tailed, paired Student’s t test was performed between time measures using a traditional design strategy and the time spent to generate the corresponding implant using implicit modeling. Significance level was set to 5%.

Moreover, to assess whether the implant generated in nTopology could satisfy criteria of surgical appropriateness, a questionnaire was administered to 5 maxillofacial surgeons. The questionnaire evaluated the characteristics for a conceptually correct computer-generated plate in terms of surgical applicability. The questionnaire was based on the attribution of a score from 0 (null) to 3 (maximal) to each item (Table 1).

**Table 1:** Structured questionnaire evaluating design appropriateness for CMF implants used in orthognathic surgery and trauma.

Items	0	1	2	3
Does the implant satisfy appropriate thickness criteria? (1.5 mm or less for orthognathic surgery)				
Are screw holes appropriately positioned in relation to skeletal buttresses?				
Does the implant include a notch to avoid injury to the infraorbital and mental nerve?				
Is the implant size compatible with the surgical access?				
Does the implant include a step around the osteotomy site?				
Do screws holes interfere with tooth roots?				
Do screw holes interfere with the inferior alveolar nerve?				
Are the plates correctly shaped over osteotomic gaps?				

Each item refers to a defined feature that accounts for usability in a surgical context.

## Results

In all cases, the bone plate generator was successful in generating the primary implant shape. Table 2 reports an overview of patients’ characteristics and software used.

**Table 2:** Patients’ characteristics in relation to their subgroups and software used.

Patient ID	Patient group	Diagnosis	Software		
			Preprocessing and point placement	Primary shape	Postprocessing
1	1	II class	Mimics and 3-matic	nTopology	3-matic
2	1	III class	Mimics and 3-matic	nTopology	Freeform + 3-matic
3	2	III class, asymmetry, condylar hyperplasia	Mimics and 3-matic	nTopology	Freeform
4	1	II class open bite	Mimics and 3-matic	nTopology	Freeform
5	1	III class	Mimics and 3-matic	nTopology	Freeform + 3-matic
6	2	II class	Mimics and 3-matic	nTopology	3-matic
7	1	III class	Mimics and 3-matic	nTopology	Freeform + 3-matic
8	2	III class, asymmetry, unilateral crossbite	Mimics and 3-matic	nTopology	3-matic
9	2	II class, maxillary contraction	Mimics and 3-matic	nTopology	Freeform
10	2	III class, bilateral crossbite	Mimics and 3-matic	nTopology	Freeform + 3-matic
			Mimics and 3-matic	nTopology	
11	3	ZMC frature	Mimics and 3-matic	nTopology	Freeform
12	3	ZMC frature	Mimics and 3-matic	nTopology	Freeform + 3-matic
13	3	Le Fort I	Mimics and 3-matic	nTopology	3-matic
14	3	ZMC + Le Fort I	Mimics and 3-matic	nTopology	Freeform + 3-matic
15	3	Le Fort II	Mimics and 3-matic	nTopology	Freeform + 3-matic
16	3	ZMC + Le Fort II	Mimics and 3-matic	nTopology	Freeform
17	3	ZMC + Le Fort I	Mimics and 3-matic	nTopology	3-matic
18	3	ZMC	Mimics and 3-matic	nTopology	Freeform + 3-matic
19	3	ZMC	Mimics and 3-matic	nTopology	Freeform
20	3	ZMC + Le Fort I	Mimics and 3-matic	nTopology	Freeform

All plates were generated under standardized conditions: minimum blend radius = 5 mm, maximum blend radius = 20 mm; the average number of points provided for maxillary plate creation = 20; average number of points provided for mandible plate BSSO creation = 8; average number of points provided for genioplasty creation = 6. Concerning screw holes, parameters were set to 1 mm for the hole radius for each plate, and all plates were designed with a standard thickness of 1 mm.

The average computational time to generate a maxillary plate implicit body once the scalar point map was provided was 5 s for the maxillary Le Fort I plate; 3 s for the mandible BSSO plate (each side was separately computed); 2 s for the genioplasty plate.

As for trauma cases, a single plate covering the inferior orbital rim, the nasal buttress and the zygomatic-maxillary buttress consisted of an average of 14 ± 3 points on each side, requiring a computational time of 15 ± 8 s. Intuitively, compared to orthognathic surgery, trauma represented a much less standardizable scenario, given the multitude of injury mechanisms and heterogeneous fracture patterns.

For orthognathic surgery and trauma, the adjustment of hole radius was almost instantaneous (up to 1 s) when the parameter was either doubled or halved, with an immediate modification of the related implicit body.

While the conversion from mesh to the implicit body was fast (on average 5 s, depending on the size of the object), the conversion from generated implicit body (i.e. plates or flanges) to triangulated meshes was more time-consuming and computationally intensive due to the re-tessellation process. The latter phase lasted, on average, 1 min and 30 s for two maxillary Le Fort I flanges and 30 s for a unilateral BSSO plate and genioplasty plate, with an accuracy set to 0.1 mm.

As for the questionnaire evaluation, all surgeons carefully reviewed the plates designed using the implicit modeling workflow and attributed a score to each item of the aforementioned list. Score 3 was the most represented (58%), followed by score 2 (36%) and score 1 (6%). Detailed results of questionnaire are reported in Table 3.

**Table 3:** Results of questionnaire administration to CMF surgeons concerning implants generated in nTopology.

	Surgeon 1	Surgeon 2	Surgeon 3	Surgeon 4	Surgeon 5	Average
Item 1	2	3	2	3	3	2.6
Item 2	3	3	1	2	3	2
Item 3	3	3	2	3	3	2.8
Item 4	2	2	2	2	3	2.2
Item 5	2	3	3	3	3	2.8
Item 6	3	2	1	3	3	2
Item 7	3	2	2	3	3	2.6
Item 8	2	3	3	3	2	2.6

Concerning the comparison of design time between traditional workflow and implicit body modeling, for all plates of orthognathic surgery and trauma, according to the study design, the workflow based on nTopology led to a statistically significant reduction in time spent during design phases. Table 4 reports in detail time measurements for implants designed using traditional techniques of implicit body modeling.

**Table 4:** Comparison in design time between implants designed using a traditional CAD workflow and implants designed combining implicit modeling and postprocessing.

Orthognathic surgery												
	Maxilla Le Fort I			Mandible BSSO right			Mandible BSSO left			Genioplasty		
	Traditional	Implicit	p-val	Traditional	Implicit	p-val	Traditional	Implicit	p-val	Traditional	Implicit	p-val
1	115	35		70	25		80	15		40	5	
2	90	30		60	15		70	20		35	5	
3	130	25		55	20		50	15		35	0	
4	80	15		70	15		55	15		30	0	
5	70	20		50	15		65	20		30	5	
6	110	20		55	35		65	30		40	5	
7	120	25		70	20		75	25		35	5	
8	100	20		60	15		75	20		40	5	
9	115	15		90	20		65	15		35	5	
10	95	20		65	25		70	20		30	0	
Mean	102.5	22.5	< 0.0001	64.5	20.5	< 0.0001	67	20	< 0.0001	35	3.5	< 0.0001
SD	18.89	6.35		11.41	6.43		9.19	4.97		4.08	2.41	

Trauma surgery												
	ZMC			Le Fort I								
	Traditional	Implicit	p-val	Traditional	Implicit	p-val						
1	90	15		150	20							
2				120	15							
3	80	10										
4				125	25							
5	65	10		110	30							
6	70	5										
7				95	15							
8				105	10							
9				110	10							
10	55	10										
Mean	72	10	0.0003	116.43	17.86	< 0.0001						
SD	13.50	3.53		17.73	7.56							

Time for implicit modeling takes into account point placement phase, nTopology calculations, STL export time and postprocessing. Time is measured in minutes at 5-min intervals.

**Figure 4:**
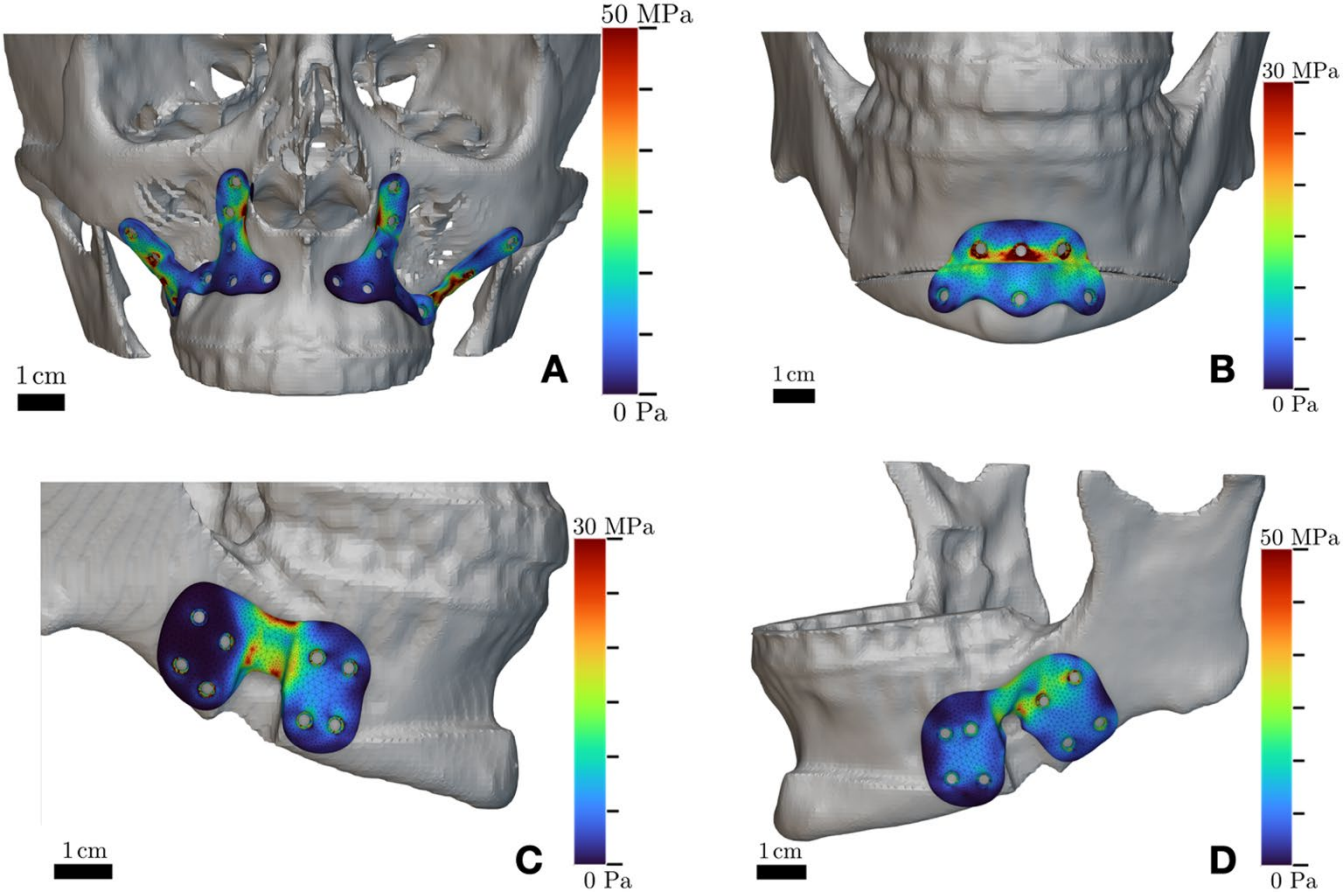
Results of FEA simulations performed in nTopology on customized bone plates for orthognathic surgery designed using implicit modeling. Stress areas are coherent with regions of maximal biomechanical load, as indicated in the color map showing Von Mises stress values. Simulation refers to patient 6.

**Figure 5:**
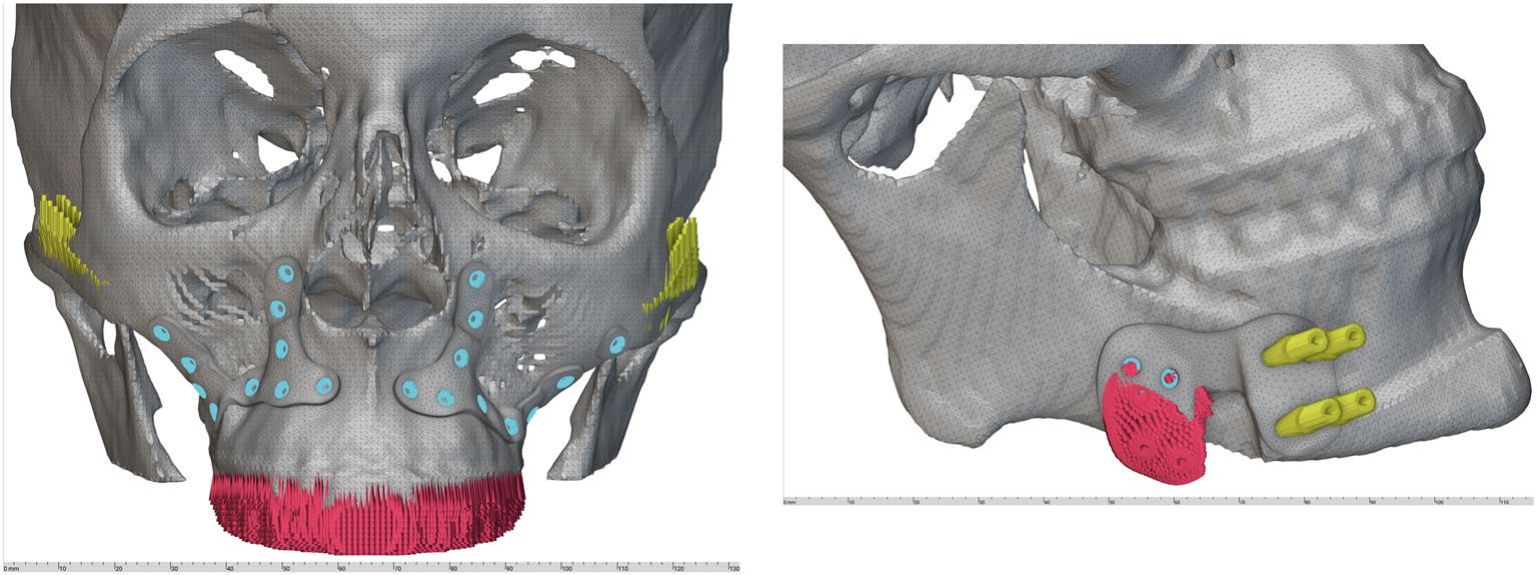
Boundary conditions applied for both the maxilla and the mandible. Displacement restraints are indicated by the red markers pointing at the fixated node. Forces are indicated by yellow arrows, pointing at the loaded node, from the direction of the force. The tie connections between implant and bone are highlighted in blue. Simulation refers to patient 6.

As for FEA simulations, the highest stresses in the implants were located around the screw holes and across the region overlying the osteotomy gap, with values ranging from a minimum of 20 to a maximum of 50 MPa. Results of FEA simulations are reported in Fig. 4, while Fig. 5 shows the definition of boundary conditions.

Table 5 reports in detail the constructive parameters for the field-driven generative design.

**Table 5:** Graphic representation of the results of FDGD for cases of orthognathic and trauma surgery according to the variation of specified constructive parameters.

Maxilla Le Fort I	Blend radius (min = 5, max = 20)	Blend radius (min = 8, max = 20)
Mean computational time: 5 s Export time: 1 m 35 s Number of points provided: 20 Blend type: sharp Hole radius: 1 mm Flange size: 0.5 mm Flange depth: 0.5 mm		
Mandible BSSO (right)	Blend radius (min = 12, max = 20)	Blend radius (min = 20, max = 20)
Mean computational time: 3 s Export time: 23 s Number of points provided: 8 Blend type: sharp Hole radius: 1 mm Flange size: 0.5 mm Flange depth: 0.5 mm		
Mandible BSSO (left)	Blend radius (min = 12, max = 20)	Blend radius (min = 20, max = 20)
Mean computational time: 3 s Export time: 20 s Number of points provided: 8 Blend type: sharp Hole radius: 1 mm Flange size: 0.5 mm Flange depth: 0.5 mm		
Genioplasty	Blend radius (min = 9, max = 20)	Blend radius (min = 12, max = 20)
Mean computational time: 2 s Export time: 10 s Number of points provided: 6 Blend type: sharp Hole radius: 1 mm Flange size: 0.5 mm Flange depth: 0.5 mm		
Mean computational time: 15 s Export time: 5 m 45 s Number of points provided: 28 Blend type: sharp Flange size: 0.5 mm Flange depth: 0.5 mm	Hole size = 1 mm	Hole size = 2 mm
	
Mean computational time: 16 s Export time: 5 m 55 s Number of points provided: 28 Blend type: sharp Flange size: 0.5 mm Flange depth: 0.5 mm	Plate thickness = 0.6 mm	Plate thickness = 1.5 mm
	

The same table reports the standardized parameters used for the selected cases.

## Discussion

Field-driven design based on implicit-modeling uses an analytical representation of fields as a foundation to optimize the structural design. Considering this novel design methodology is very recent, there are no clinical reports in the literature on field-driven design implementation to fabricate biomedical implants.

Topological optimization, which can be considered a precursor of field-driven design, has been applied several times in the medical field. These applications are restricted to mechanical engineering, especially for the design of lattice structures, where finite element analysis (FEA) can be used to evaluate properties like stiffness and deformability associated with the lattice patterns, which are modified according to the results of the simulations^12–14^. Topological optimization can thus adjust these patterns based on the findings of biomechanical testing, as demonstrated by Cantaboni et al.^13^.

While topological optimization typically uses stress–strain fields calculated from iterative FEA and then seeks optimization to provide structural integrity, reducing the amount of material used^15^. The fields considered in the more general field-driven approach can be different, e.g. thermal, magnetic, and flow-velocity. The optimization process seeks a structure that optimizes these different fields (simultaneously) and any additional constraints. In the current work, the underlying field used is a distance field created from the mesh representation of the anatomical structures. This field represents the distances between each point in space and the closest nearby points on a surface. Therefore, this approach can achieve a design that matches the underlying bone structure, represented by a distance field. Moreover, it enables easy modifications to the designed structure while maintaining geometric conformity and adhering to the imposed constraints.

Given such premises, FDGD represents a promising approach to optimize time and resources and can also be applied in the medical field, where a geometrical field based on the underlying implicit anatomy is used to create a precursor of the final customized implant generatively.

Our proposed workflow demonstrated the successful integration of implicit bodies into current virtual surgical planning software, supporting a more efficient and time-saving procedure to generate plates for maxillofacial surgery.

Compared to traditional explicit modeling based on CAD operations, such as curve creation, face extrusion, Booleans and surface offset, the field-driven design offers an intelligent trade-off between flexibility and simplicity. Applying this generative methodology can significantly improve implant development processes, achieving unprecedented efficiency. For instance, a field allows instantaneous control over several parameters, including the location and radius of each screw hole, plate thickness and surface texture roughness.

Conventionally, in an explicit modeling sequence, the surgeon first defines the position of screw holes in the anatomical virtual replica. Subsequently, multiple operations occur, including defining the outline of the plate, extruding the template body from the anatomical template, subtracting it from the plate, and then subtracting screws from the plate to achieve the final holes. All these stages are time-consuming and might last several hours. Moreover, traditional design is non-reversible: when one mm-radius screw is subtracted from the plate, it is not feasible to modify the same screw hole to a 0.5 mm radius unless there is the original, non-subtracted plate hole kept as a backup replica. Moreover, Boolean operations performed on high-resolution geometries are often complex. In many cases, geometrical artifacts can be avoided only with meticulous mesh curing and topological checking before executing the operation.

Using parametric modeling can partly address the aforementioned problems by allowing for the modification of geometrical properties. Software such as PTC Creo, Solidworks and Catia are parametric, are based on conventional geometries and rely on curve-based geometries such as IGES and STEP files. These geometries are not well-suited for complex anatomical shapes of the craniofacial region, creating multiple patches to approximate the organic skeletal surface^16^. This often results in computational failures and unsustainable processing times, which can be addressed through implicit modelling. Moreover, parametric packages lack generative features and are mainly used in mechanical engineering. Their utilization in medical modelling is uncommon, as demonstrated by the authors in a recent, extensive systematic review^17^.

All these operations are simplified in FDGD. While executing operations in extremely short amounts of time, including almost immediate Booleans, and preserving a great anatomical detail when skeletal surfaces are converted to implicit bodies, FDGD at the same time maintains control over parameters that can be repeatedly modified. For instance, the same plate can be tested with screws of different sizes without having to design a new object.

One of the key factors for mass personalization in surgery, namely, the use of patient-specific solutions for all surgical procedures, is the time to delivery of the implant. Currently, the time required for implant design is a major obstacle. In cases where sudden modifications are needed, it is challenging to step back through all design phases and revert the original plan to the new configuration. While orthognathic surgery allows a more permissive timeline from planning to surgery, trauma surgery is more time sensitive. Thus, the implementation of personalized implants is significantly limited by time constraints. However, the increased adoption of FDGD specifically developed for medical applications may significantly reduce the time required to design customized implants.

The bone plate generator module incorporated in nTopology provides a preliminary estimation of the potential of FDGD in maxillofacial surgery. However, it is limited to plates of the linear structure whose shape is determined by the position of a guiding point map. A further improvement will be represented by novel software implementations that enable to design of alternative implant types, including those for orbital and cranial reconstruction as implicit bodies. Moreover, implicit modeling will allow to model of patient-specific implants that align with the outcomes of FEA analysis^18^, allowing for the optimization of the topological design based on the shear stress and tension the implant is anticipated to endure. This is in line with the “mechanobiological” concept described by Ruf et al.^19^, yet this would require additional studies, since this aspect was not developed in this paper.

Limitations of this study include its retrospective design, as cases were selected from a cohort of patients who had already undergone surgery with traditional plates. Prospective enrollment of patients who receive implants designed through either FDGD or conventional methods and evaluating the difference in production time and final quality would be a necessary improvement to establish evidence on the importance of this new technology in the medical field.

As Fig. 2 shows, implants generated through FDGD are apparently more cumbersome, as the software builds the field geometry around the predefined hole points. Although this feature can be parametrically modified by varying the blend radius, the overall primary implant shape is cumbersome and needs trimming to maximize its compatibility with the anatomical recipient site. Conversely, currently available personalized implants are designed to be minimally invasive, with thin bars connecting the screw holes. However, a slight extension of the plate area might contribute to future innovative design, which may embed lattice structures to facilitate osteointegration, as well as FEA-oriented geometries that may help to stabilize the mechanical load on bone segments.

Moreover, the bone plate generator module is very preliminary in its conception, and the software is not able to recognize that the mandibular gap is an empty zone and that the plate should be conformed as a bridge bypassing it, rather than wrapping it. Thus, plates need to be edited in 3-Matic or Geomagic Freeform to improve their anatomical compatibility. But still, field-driven design considerably simplifies the design process, as the implant custom-fitted surfaces, including screw holes of the desired diameter, are automatically generated, and with minimal mesh editing the designer can achieve a primary implant shape. Moreover, as FEA data suggest, the primary plate design generated after placing points needs a further optimization to fulfill mechanical requirements, and this should be acknowledged as a limitation at this stage of development of nTopology for medical purposes. An additional limitation is that the evaluation of time reduction using FDGD is just an estimation, however reduction in time might be considerable, as well as the easiness of managing parametrically the diameters of the screw holes, which can be interactively and instantaneously modified. The same is true also for the mental foramen, which nTopology is not able to recognize, thus mesh editing must include the creation of a notch surrounding this foramen to avoid compression on the mental nerve. Another critical point is represented by thin bones, such as the anterior maxillary wall: nTopology is not able to recognize anatomy, and once the guiding points are assigned, the software performs a front-face and back-face wrapping around the piriform notch. Yet, it is very easy for the user to clear the duplication of geometry behind the bone, at the same time preserving the plate structure where needed, with the hole diameter and plate thickness that can be parametrically modified. In particular, FDGD showed its potential for elaborating primary implant shapes but the study cannot demonstrate at this stage the complete feasibility of the method for the final design of custom maxillofacial devices. Indeed, this workflow still needs further improvements to ensure a correct performance and function of devices. In addition, this specific FDGD application consider only distance as a field, but it should be integrated with computational information including also the correct simulation of mechanical loads. At its current stage, as demonstrated by FEA, FDGD might be not sufficient to achieve an appropriate final design, as it would still rely on further optimization of the geometries.

However, the preliminary results shown in this paper suggest that FDGD has the potential to simplify the highly complex workflow of designing customized plates, which currently requires specialized expertise possessed by only a small fraction of clinicians. This could facilitate more healthcare institutions to design personalized implants in-house, thereby reducing the overall planning time and facilitating the widespread of customized craniofacial implants in surgery, given the well-demonstrated benefits in terms of surgical accuracy and ease of placement.

## Conclusions

In summary, this work discusses a novel methodology to design patient-specific implants that could potentially replace current workflows based on traditional design features in the coming years. It aims at shedding light on the clinical applications of an innovative technique to enhance customized implants' design possibilities. In its attempt, this study still has several limitations in methodology and showed the potential for the elaboration of the primary implant shape for customized maxillofacial plates. Future advancements of FDGD by software companies and the development of more clinically oriented packages will enhance their integration with current clinical workflow.

## Data Availability

The datasets generated during and/or analysed during the current study are available from the corresponding author on reasonable request.
